# Bacterial Analysis of the Whole Blood in Chinese Healthy Donors Using 16S rDNA–Targeted Metagenomic Sequencing

**DOI:** 10.1155/2024/6635560

**Published:** 2024-10-16

**Authors:** Jingjing Zhang, Yanmin He, Chen Chen, Wei Hu, Ji He, Yanling Ying, Faming Zhu

**Affiliations:** Institute of Transfusion Medicine, Blood Center of Zhejiang Province, Hangzhou, China

**Keywords:** 16S rDNA, blood components, blood microbiome, Chinese blood donors, whole blood

## Abstract

**Background:** The presence of bacteria in the blood of healthy individuals remains controversial. This study explored the comprehensive bacterial profiles and specific biomarkers in different components of healthy Chinese blood donors.

**Methods:** A total of 5230 whole blood (WB) specimens were collected. Among them, 5200 random samples were pooled into 26 mixed samples for bacterial profile analysis. The remaining 30 random samples were divided into 4 groups based on components: WB, plasma, red blood cells (RBCs), and buffy coat (BC). Subsequently, the amplicons of the bacterial 16S rDNA V3–V4 fragments were sequenced to measure the diversity and composition of the bacteria using next-generation sequencing.

**Results:** The bacterial DNAs in the blood primarily originated from the Proteobacteria phylum. A total of 301 species of bacterial DNA were found in blood specimens, with 46 species being present among all groups. A significantly higher abundance of bacterial DNA was found in the plasma and RBCs compared to those in BC and WB. However, the plasma and RBC groups showed significantly higher species diversity and richness compared to the BC and WB groups. In addition, the WB group had a significantly different community structure and composition compared to the plasma and RBC groups but was similar to the BC group.

**Conclusion:** The presence of bacterial DNA fragments was confirmed in blood from healthy Chinese donors. The bacterial DNA fragments enriched in plasma showed the highest diversity, followed by RBC, WB, and BC. These results provide a foundation for further research on the microbiome in the blood of healthy individuals.

## 1. Introduction

Human blood has traditionally been considered to be a sterile environment due to the lack of proliferating microorganisms [1]. However, the presence of a microbiome in the blood of donors implies the risk of transfusion-transmitted bacterial infection by platelet, red blood cell, and/or cryoprecipitate component transfusion [2, 3]. Despite a reduction in transfusion-transmitted diseases due to the improvement of the detection technology and capability, serious adverse events are still encountered [4]. Interestingly, the presence of a healthy human blood microbiome (HBM) has been increasingly accepted, supported by the growing evidence showing the existence of a blood microbiome in healthy individuals. Despite the presence of the bacteria in healthy blood, no symptoms of infection were found [5–7]. Notably, the origins, identities, and functions of the healthy HBM in blood donors remain unelucidated. Païssé et al. [8] reported the significant differences in the microbial spectrum present in different components of blood from French blood donors. However, the data from various blood donors in different regions are limited. Furthermore, data on the incompetent blood components of healthy blood donors in the Chinese population have not yet been reported. Moreover, the selection criteria and detection agents for blood donors vary across different countries, and the residual risks of blood donation also vary in different countries. Therefore, determining the blood microbial distribution in different populations or regions may improve the safety of blood transfusion. In addition, transfusions of blood-containing microbiomes may cause dysbiosis in patients. Blood microbiome dysbiosis is associated with noninfectious diseases, including Parkinson's disease, liver fibrosis, diabetes, and gastric cancer. [9–14]. Therefore, exploring the microorganisms present in whole blood and different blood components could help to decrease the risk of transfusion-transmitted bacterial infection and could even identify blood markers for disease prevention and prognosis.

Red blood cells and plasma products are not routinely subjected to bacterial culture testing. Therefore, transfusion-transmitted bacterial infection may occur, which may even be life-threatening. Due to the high diversity and extremely low abundance of the microbiota in the blood of healthy individuals, they cannot be detected by conventional bacterial culture methods. Molecular techniques, such as quantitative polymerase chain reaction (qPCR) and next-generation sequencing (NGS) [15–17], are rapid and offer high sensitivity and have been widely used to detect the microorganisms of some patients [18–20]. Microbial 16S rDNA as a mature genetic marker is widely used in the identification and classification of bacteria [21]. Therefore, metagenomic analysis based on 16S rDNA was preferably used to analyze the blood microbiota in healthy individuals by the NGS method [22].

In this study, 16S rDNA–targeted metagenomic sequencing was performed to detect the bacterial DNA composition and identify the bacterial biomarkers of different blood components (including WB, RBCs, plasma, and buffy coat). Our study yielded some different results compared to previous reports [7, 8]. The results facilitate a more comprehensive and diverse characterization of the blood microbiome in healthy individuals, improving the safety and effective supply of blood products.

## 2. Materials and Methods

### 2.1. Blood Specimen Collection

Blood samples were collected from a total of 5230 healthy donors in the Blood Center of Zhejiang Province, China. The project was approved by the Ethics Committee of the Blood Center of Zhejiang Province (approval no. WKJ-ZJ-1815). This study was conducted in accordance with the Chinese National Standard for Whole Blood and Component Donor Selection Requirements (GB18467-2011). Written informed consent was obtained from all participants. All donors were required to fill out the health consultation form before donation; subsequently, all donors received a health history questionnaire, a brief physical examination, and rapid predonation testing according to the guidelines for blood donation in China. The donors who did not conform to the standard of China or received certain medical treatments such as antibiotics were excluded from donation. Then, WB was collected in one aseptic tube with EDTA anticoagulant from each healthy donor after the topical disinfection of the skin. HBsAg, anti-HCV, anti-HIV1/2, anti-TP, HBV DNA, HCV RNA, and HIV RNA were negative for all these donors. Sterilized water was used as a negative control sample

### 2.2. Preparation for Blood Components

**Figure 1:**
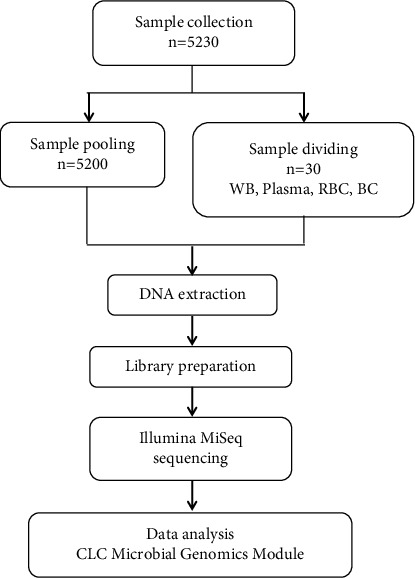
Schematic representation of the workflow for blood sample preparation, genomic extraction, library preparation, sequencing using Illumina MiSeq, and metagenomics analysis using the CLC Microbial Genomics Module.

WB samples were collected directly from each donor. Thereafter, 5200 samples of WB were mixed in a ratio of 200:1, yielding a total of 26 WB mixed samples. The remaining 30 WB specimens were divided into four groups according to blood components, including the WB group (WB), plasma group (plasma), RBC group (RBC), and BC group (BC) (Figure 1). The blood components were prepared from the WB after centrifugation at 2500 × *g* for 15 min at 4°C. All specimens were operated with aseptic techniques under a class II biologic safety cabinet and immediately frozen in liquid nitrogen and then stored at −80°C freezer until DNA extraction.

### 2.3. DNA Extraction From WB, Plasma, RBC, and BC Specimens

DNA was extracted from mixed WB and individual blood component (WB, plasma, RBC, and BC) specimens using a MagNA Pure LC DNA Isolation Kit III (Roche Diagnostics Inc, Indianapolis, IN, USA) according to the manufacturer's instructions. The quality and quantity of DNAs were detected by an ultraviolet spectrophotometer (Multiskan GO, Thermo Scientific, Waltham, MA, USA). The final DNA concentration was adjusted to 10 ng/μL.

### 2.4. 16S rDNA Amplicon and Sequencing

The QIAseq 16S/ITS panel was used to amplify the V3–V4 regions of the 16S rDNA, and the library was prepared according to the manufacturer's instructions (Qiagen Company, Hilden, Germany). In brief, the specific primers provided in the panel were used for the amplification of the V3-V4 region fragments. After the first purification, a secondary amplification was conducted to add universal adapters. The final sequencing libraries were obtained after the purification and were quality checked by the Agilent 4200 TapeStation Instrument (Agilent Technologies, Inc, Santa Clara, CA, USA) to ensure an average length of fragment of about 600 bp. All samples were homogenized to the same concentration and pooled in equal quantities into one tube. The library was denatured with 0.2 mol/L NaOH , diluted to a final concentration of 10 pmmol/L, and then sequenced using an Illumina MiSeq instrument and a v3 Reagent Kit (600-cycle; Illumina Inc, San Diego, CA, USA). The 16S rDNA original data were saved in the fastq format by the software in the MiSeq instrument.

### 2.5. Operational Taxonomic Units' Analysis and Species Annotation

Data from the bacterial classification were analyzed using the CLC Microbial Genomics Module (version 21, Qiagen Company, Hilden, Germany). After removing the linker sequence, quality control was performed based on the barcode and primer sequences to obtain a valid sequence (devoid of repetitive and poor-quality reads). The background sequences were removed from the human genome and the sequences were clustered into “Operational Taxonomic Units” (OTUs) based on 97% similarity using Greengenes (V13) [20, 23] ribosomal database reference dataset. Then, a set of the same sequences was subjected to species annotation and classification based on NCBI Taxonomy databases. Bacteria showing a combined abundance of less than 100 were eliminated.

### 2.6. Species Composition Analysis

The species compositions of different groups were visualized with stacked bar charts, sunburst view, and heat maps. The OTU abundance tables containing the chimeras provided the abundance of the OTU or chimeras at each site, as well as the total abundance for all specimens. The OTU abundance table was visualized as a stacked bar. As the classification level of species was continuously refined (kingdom, phylum, class, order, family, genus, and species), a growing number of species categories was found. In order to ensure the visual effect of the figure, only the phylum level and species level were shown. Subsequently, TMM (trimmed mean of *M* values) normalization was performed for the abundance of species using the create heat map for abundance table tool, followed by a *z*-score normalization to make features comparable. The hierarchical clustering groups' features were clustered by the similarity features of their genomes on a set of samples. The top 20 species in each specimen from different groups were compared, as well as the species common among all groups.

### 2.7. Alpha and Beta Diversity Analysis

According to the normalized OTU abundance, alpha diversity indices (total number, Shannon entropy, Simpson's index, and Chao 1 bias-corrected) were calculated using CLC Genomics Workbench software (version 21). The alpha diversity indices among the different groups were analyzed by the Kruskal–Wallis H test. Beta diversity was analyzed for the similarity of bacterial community structure among different groups [24], and the distance matrix was calculated by principal coordinate analysis (PCoA) using CLC Genomics Workbench software based on Jaccard and Bray–Curtis' methods.

### 2.8. Statistical Analysis

Statistical analysis was conducted using GraphPad Prism 5 (GraphPad, CA), and *p* < 0.05 was considered as statistically significant. Microorganism features for distinguishing the blood microbiota were identified using the linear discriminant analysis (LDA) effect size (LEfSe) method [25] with the alpha parameter of 0.05 for both pairwise Wilcoxon and factorial Kruskal–Wallis rank sum tests with a threshold of 2.0 for LDA.

## 3. Results

### 3.1. Bacterial Metagenomic Analysis of Mixed Blood Samples From 5200 WB Donors

**Figure 2:**
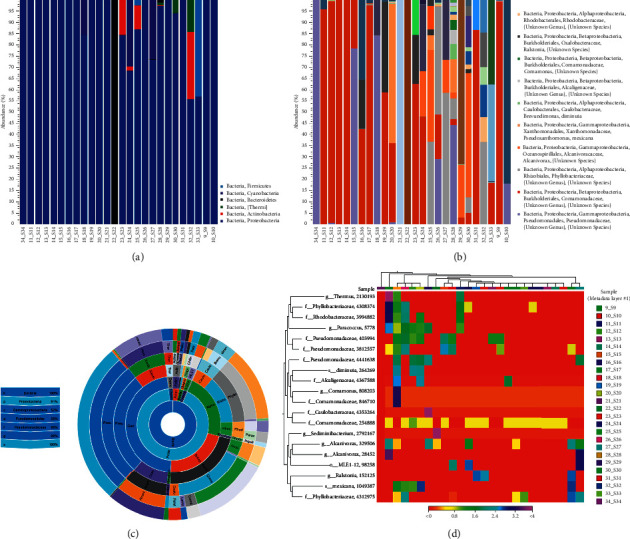
The microbiota species and abundance analysis of mixed whole blood from 5200 whole blood donors. (a) Stacked bar of the microbial community at the phylum level. (b) Stacked bar of the microbial community at the species level. (c) Sunburst view of the microbial community showing all taxa belonging to the kingdom bacteria. (d) Heat map analysis of microbiota in 5200 whole blood donors.

The overall microbial profile of the WB from blood donors was analyzed using a large-scale mixed sample. A total of 38 species of 16S bacterial DNA were found in the mixed WB. Proteobacteria was the most dominant phylum, accounting for 91% of all bacteria from mixed WB samples, followed by Actinobacteria (Figures 2(a), 2(b), and 2(c)). At a deeper taxonomic level, Pseudomonadaceae, Comamonadaceae, Phyllobacteriaceae, and *Alcanivorax* were significantly enriched in the mixed WB. This analysis further visualized the top 20 species' composition in the combined abundance of mixed WB in the form of a heat map. Genes or specimens with similar expression patterns would be classified into one category and closer together in the evolutionary tree (Figure 2(d)).

### 3.2. Similarities and Differences in Microbiota Distribution Among Different Blood Components of Chinese Healthy Blood Donors

**Figure 3:**
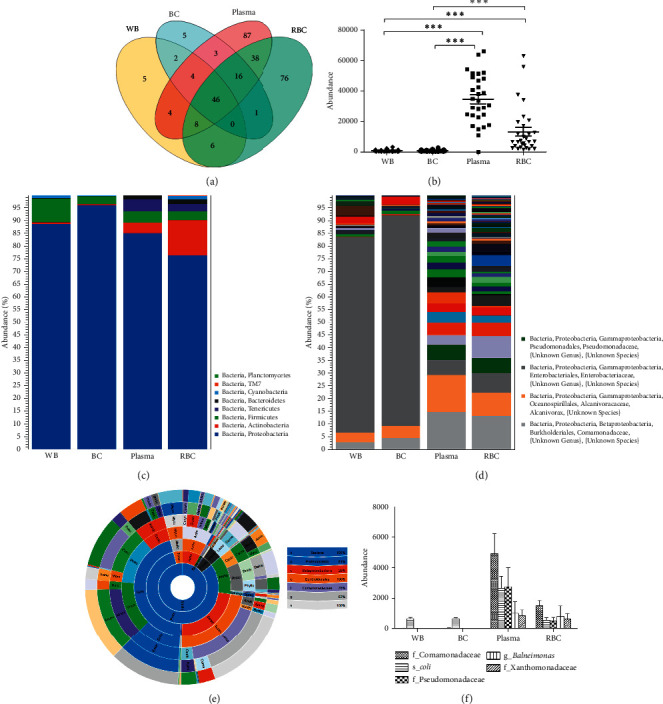
The microbiota species and abundance analysis of WB, BC, plasma, and RBC groups from whole blood donors. (a) Venn diagram. (b) Statistical analysis of bacterial abundance among different blood components. (c) and (d) Stacked bar of the microbial community among WB, BC, plasma, and RBC at the phylum level and species level, respectively. (e) Sunburst view of the microbial community showing all taxa belonging to the kingdom bacteria. (f) Observed abundance of dominant bacteria among WB, BC, plasma, and RBC. Abbreviations: BC, buffy coat; RBC, red blood cell; WB, whole blood. ⁣^∗∗∗^*p* < 0.001.

**Figure 4:**
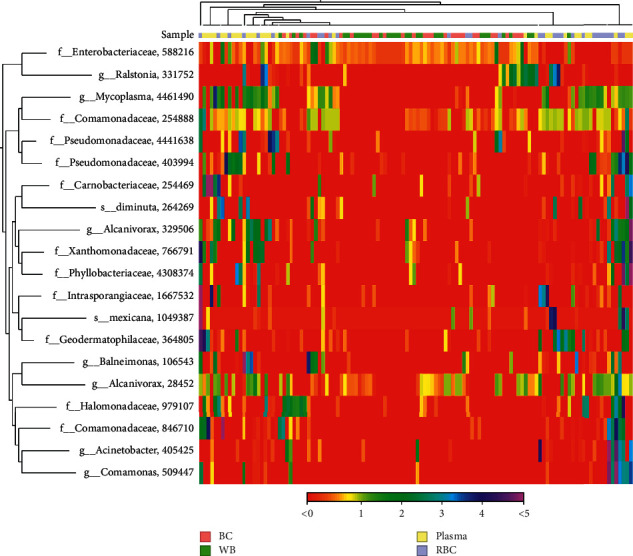
Heat map analysis of top 30 microbiota in WB, plasma, RBC, and BC groups. Abbreviations: J, plasma; Q, whole blood; R, red blood cells; W, buffy coat.

The specific characteristics of the microbiota flora in blood were determined by 16S rDNA-targeted metagenomic analysis of the four blood components. The species composition of the four blood components is shown in Figure 3. A total of 301 species of bacterial DNA were found in the blood samples. Plasma specimens contained the most, i.e., 206 species, followed by the RBC group (191 species), the BC group (77 species), and the WB group (75 species). A total of 46 species were shared among all groups (Figure 3(a)). A significantly higher abundance of bacterial DNA was observed in the plasma (34,548 ± 15,982) and RBC (13,256 ± 15,250) groups (*p* < 0.001) compared to those in the WB (952 ± 669) and BC (861 ± 497) groups (Figure 3(b)). A total of eight phyla (Proteobacteria, Actinobacteria, Firmicutes, Tenericutes, Bacteroidetes, Cyanobacteria, TM7, and Planctomycetes) and dozens of genera were found in the four groups (Figures 3(c) and 3(d)). Figure 3(d) indicates that a significantly larger number of bacterial DNA species was found in the plasma and RBC groups than in the BC and WB groups. Proteobacteria was the most dominant phylum, accounting for 81% of all bacteria across the four groups, and at least 75% of the bacterial composition of each blood component (Figures 3(c) and 3(e)). At a deeper taxonomic level, Comamonadaceae, Pseudomonadaceae, and *coli* were significantly enriched in the plasma group; Comamonadaceae, *Balneimonas*, and Xanthomonadaceae were significantly enriched in the RBC group; *coli* was significantly enriched in the BC and WB groups (Figure 3(f)). This analysis further visualized the top 20 species composition in the combined abundance of each specimen in the form of a heat map (Figure 4). The top 20 bacteria had higher expression levels in the plasma and RBC groups than in the WB and BC groups.

### 3.3. The Bacterial DNA Species Diversity and Microbial Community Structure of the BC and WB Groups Were Significantly Different From That of the RBC and Plasma Groups

**Figure 5:**
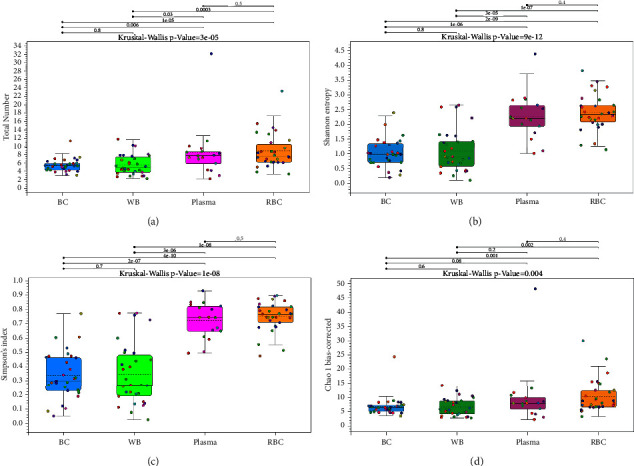
Alpha diversities shown in a box plot. (a) Total number. (b) Chao 1 bias-corrected. (c) Simpson's index. (d) Shannon entropy.

The alpha diversity indices (total number, Chao 1 bias-corrected, Simpson's index, and Shannon entropy) were used to evaluate species diversity (Figure 5). Compared with the RBC group, the total number (Figure 5(a)) and the Chao 1 bias-corrected (Figure 5(b)) of the BC and WB groups were all significantly reduced (*p* < 0.001). In addition, the total number (Figure 5(a)) of the BC group was significantly lower (*p* < 0.001) than that of the plasma group. Moreover, Simpson's index (Figure 5(c)) and Shannon entropy (Figure 5(d)) of the RBC and plasma groups were all significantly higher than the BC and WB groups (*p* < 0.001). Significant differences in species diversity and richness were found in the plasma and RBC components compared to the BC and WB components.

**Figure 6:**
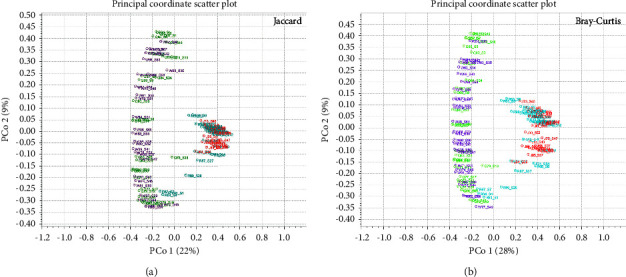
Beta diversity results seen as 2D PCoA, with coloring performed according to taxonomic abundance values. (a) Jaccard. (b) Bray–Curtis. Abbreviations: J, plasma; Q, whole blood; R, red blood cells; W, buffy coat.

The beta diversity among the WB, plasma, RBC, and BC microbial communities was analyzed using two assessment methods (Jaccard and Bray–Curtis) (Figure 6). The results demonstrated that PCo1 explained 22% (Jaccard) (Figure 6(a)) and 28% (Bray–Curtis) (Figure 6(b)) of the total variation, while PCo2 explained 9% (Figures 6(a) and 6(b)). Therefore, PCo1 analysis was preferentially used to assess the taxonomic relationship of these four groups. Overall, the PCo1 of the WB and BC specimens was almost the same; the PCo1 of the plasma and RBC groups was similar. Moreover, the PCo1 of the WB group exhibited a certain distance from the plasma and RBC groups but was similar to the BC group. Thus, the microbial community structure and composition of the WB group was significantly different from the plasma and RBC groups but similar to that of the BC group.

### 3.4. Differential Bacterial Profiles Among Blood Components and Biomarker Identification

**Figure 7:**
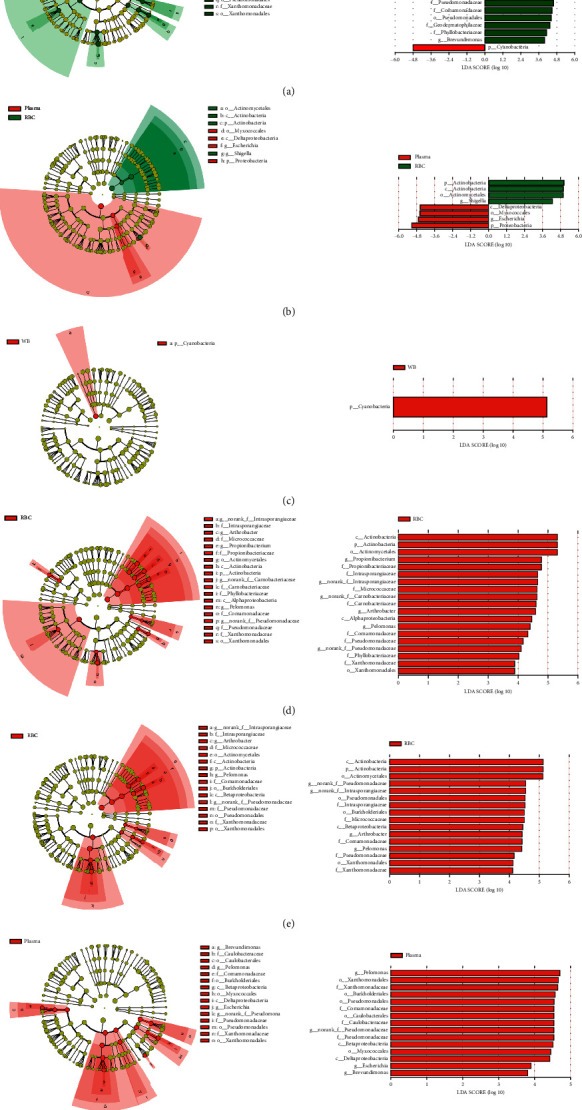
Linear discriminant analysis (LDA) effect size (LEfSe) analysis revealed significant bacterial differences in the WB, plasma, RBC, and BC groups. The LDA scores (log 10) > 2 and *p* < 0.05 are listed. (a) WB vs plasma. (b) Plasma vs RBC. (c) WB vs BC. (d) WB vs RBC. (e) RBC vs BC. (f) Plasma vs BC. Abbreviations: BC, buffy coat; RBC, red blood cells; WB, whole blood.

Statistically significant differences in microbial communities among the WB, plasma, RBC, and BC groups were analyzed by LEfSe analysis. Cladograms of the taxa with LDA values > 2.0 are depicted in Figure 7. Significant differences in the community compositions were observed among the four groups. Notably, 19 differential bacterial DNA species were found between the plasma and WB groups; Actinobacteria and other 17 specific bacteria species were present in the plasma group, while only *Cyanobacteria* was found in the WB group, as shown in Figure 7(a). Moreover, Actinobacteria, *Actinomycetales*, and *Shigella* were enriched in the RBC group, while Proteobacteria, *Escherichia*, *Myxococcales*, and *Deltaproteobacteria* were enriched in the plasma group, as displayed in Figure 7(b). Between the remaining pairwise comparisons, only one differential bacterial DNA species was observed. For instance, one phylum (*Cyanobacteria*) distinguished the microbial communities between the WB and BC groups, as shown in Figure 7(c). Nineteen and sixteen bacterial DNA species, including Actinobacteria, distinguished the microbial communities between the WB and RBC groups (Figure 7(d)), and the BC and RBC groups (Figure 7(e)), respectively. Compared with the BC group, *Pelomonas* and Xanthomonadaceae were enriched in the plasma group (Figure 7(f)).

## 4. Discussion

In recent years, multiple studies have reported the blood microbiome in healthy donors [26]. However, the origin and function of the healthy HBM across different regions and populations require further research. This study investigated the bacterial distribution of different blood components in healthy Chinese blood donors. A certain amount of 16S rDNA was detected in blood from healthy donors, showing consistent results with those previously reported [7, 27]. The experiments described the existence of low-biomass microbiomes prone to contamination from external sources, which may affect the accuracy of blood microbiome analysis in healthy donors [28]. In order to avoid any risk of contamination between specimens or from the experiments in our study, all open procedures were performed with aseptic techniques under a class II biologic safety cabinet. In addition, all blood specimens were collected from healthy donors after standard skin topical disinfection; the disposable consumables and reagents were also specifically optimized to reduce and control contaminant bacterial DNA. Our data confirmed the presence of 16S rDNA fragments in the blood of healthy donors.

According to the results of our study, Proteobacteria was the most dominant phylum in 5200 mixed WB samples, followed by Actinobacteria and Firmicutes in the blood component concentration. The composition of the blood microbiome of WB donors in this study was similar to the results of previous reports [8, 29]. However, whether the blood microbiomes are exploiting a viable ecological niche or only represent transient residents in the blood remains unknown. The source, characteristics, and functional effects of these bacteria require further research. Païssé et al. [8] found that Proteobacteria (more than 80%), Actinobacteria, and Firmicutes were the dominant flora in the blood of French blood donors. Their results indicated that the detection of these bacteria in the blood components of healthy blood donors was likely to be caused by incomplete skin disinfection. The data from Gao et al. [30] also clarified that Actinobacteria, Firmicutes, and Proteobacteria were the main skin bacteria present in the volar surface of the forearm for healthy individuals in the United States. However, the healthy HBM may originate from the gut microbiomes and oral microbiomes, resulting from bacterial translocation mechanisms; another possible source is the maternal origin [8, 20, 31, 32].

Furthermore, differences in bacterial species expression and abundance were observed between different mixed groups. This may be attributed to the differences between individual WB donors. The peripheral blood of 30 WB donors was divided into four groups to clarify whether bacterial species and abundance differ between different blood components. The abundance, diversity, and heterogeneity of microbial composition exhibited significant differences among WB and each blood component in our study. Alpha diversity is an index reflecting the variety of microbial species in blood specimens, with a higher alpha diversity indicating higher abundance [33]. This alpha diversity indices analysis revealed that the abundance and diversity of bacterial composition in the plasma and RBC groups were significantly higher than in the BC and WB groups. In addition, beta diversity is an index reflecting the heterogeneity of microbiota among the specimens in each group [34]. Higher beta diversity is indicative of larger compositional differences in the blood microbiota among certain specimens. PCoA mainly uses specimen distance information for calculation and dimensionality reduction graphics display, so the results are more focused on reflecting the dissimilarity distance between specimens [22]. PCoA analysis revealed that the structure of the blood microbiota of the BC and WB groups was similar and clustered together, while similar structures of blood microbiota were found in the RBC and plasma groups. Bacteria can bind to the surface of or enter RBCs, which plays an essential role in the immobilization and capture of various bacteria in immune response and antibacterial defense [8]. In this case, the RBC group contained a non-negligible amount of bacterial DNAs, which were significantly higher than the WB and BC groups, while also possibly containing a higher number of bacterial species compared to the BC group. Therefore, RBCs may act as carriers of bacterial pathogens, causing transfusion-transmitted bacterial infections. New pathogen reduction or screening methods are needed to reduce the risk of persistent infection associated with RBC blood transfusion. However, both bacterial DNA and species were more abundant in the plasma group compared to the RBC group, and the abundance of bacterial DNA in the BC group was not the most enriched in this study, which differed from the previous report [7, 8]. This may be attributed to the interference of the human genomic DNA for amplification and the limitation of output when using the Illumina MiSeq instrument for NGS. Therefore, increasing the sequencing depth by changing the NGS output and decreasing the human genomic DNA will benefit the analysis of the abundance of bacterial DNA in the BC group. The statistical differences between microbial communities were analyzed among the WB, plasma, RBC, and BC groups; OTUs were compared with the LEfSe analysis. Bacteria showing significant differences (LDA > 2.0; *p* < 0.05) between the groups can be used as potential biomarkers to distinguish different blood components in the future.

The data in this study indicated that the blood of healthy donors was not sterile, which was consistent with previous reports [28, 35]. Therefore, elucidating the microbiota in the blood of healthy individuals holds significance in planning preventive measures to reduce the risk of infection during blood transfusion. Theoretically, microorganisms in the blood can be transmitted through blood transfusion. Studies have shown that daily activities such as chewing and tooth brushing facilitate the translocation of bacteria into the blood [13]. In addition, periodontitis is an inflammatory condition in which bacteria may gain access to the bloodstream [28, 36]. However, periodontitis is currently not an exclusion criterion for blood donation. Therefore, collecting blood from such donors will pose a certain risk to the safety of blood transfusion. Moreover, the 16S rDNA of the pathogen detected by NGS technology mostly reflects the nucleic acid fragment of the pathogen, which may correspond to free bacteria or free bacterial DNA produced by immune degradation. Therefore, whether the detected are live or dormant cannot be determined. Future improvements in blood supply screening with more sensitive and detailed molecular assays will help improve the safety of blood transfusion. However, due to the high sensitivity of molecular biology techniques, blood-containing bacterial DNA concentrations that may not be clinically relevant could be discarded. We suggest that this approach be used to exclude certain units intended for transfusion in high-risk individuals, such as immunocompromised patients.

Nevertheless, the limitations of the present study should be acknowledged. First, the factors related to bacterial localization and the proportion of DNA derived from living, nonviable, or dormant bacteria could not be determined. A thorough understanding of the blood microbiome of healthy blood donors will require detailed studies in the future. Moreover, only a small number of blood donors have been evaluated in this study, which should be increased in future studies. Furthermore, our study is a cross-sectional study. Longitudinal studies are needed to confirm the microbiota in the blood at different times and from different regions. In addition, the storage condition of platelets is more suitable for bacterial growth. However, the main subjects of this study were WB donors. Our future research may focus on the bacterial analysis of platelets from apheresis platelet donors.

In this study, a metagenomics method based on NGS technology was used to detect 16S rDNA of different blood components in Chinese healthy blood donors. The results of this study indicated the presence of bacterial sequence fragments in the blood of healthy blood donors. Bacterial DNA and species diversity were most abundant in the plasma component and least abundant in the BC component. A thorough understanding of the blood microbiome of healthy blood donors is essential for the safety of blood transfusion, which relies on sensitive and specific methods to detect bacteria in the blood.

## Data Availability

The data used to support the findings of this study are openly available in NCBI Sequence Read Archive at https://www.ncbi.nlm.nih.gov/bioproject/PRJNA809084/, reference number: PRJNA809084.
